# Supplementary figures and images for: SPI-1 virulence gene expression modulates motility of Salmonella Typhimurium in a proton motive force- and adhesins-dependent manner
Source: PLoS Pathog. 2023 Jun 14;19(6):e1011451. doi: 10.1371/journal.ppat.1011451 (PMC10298799; doi:10.1371/journal.ppat.1011451)

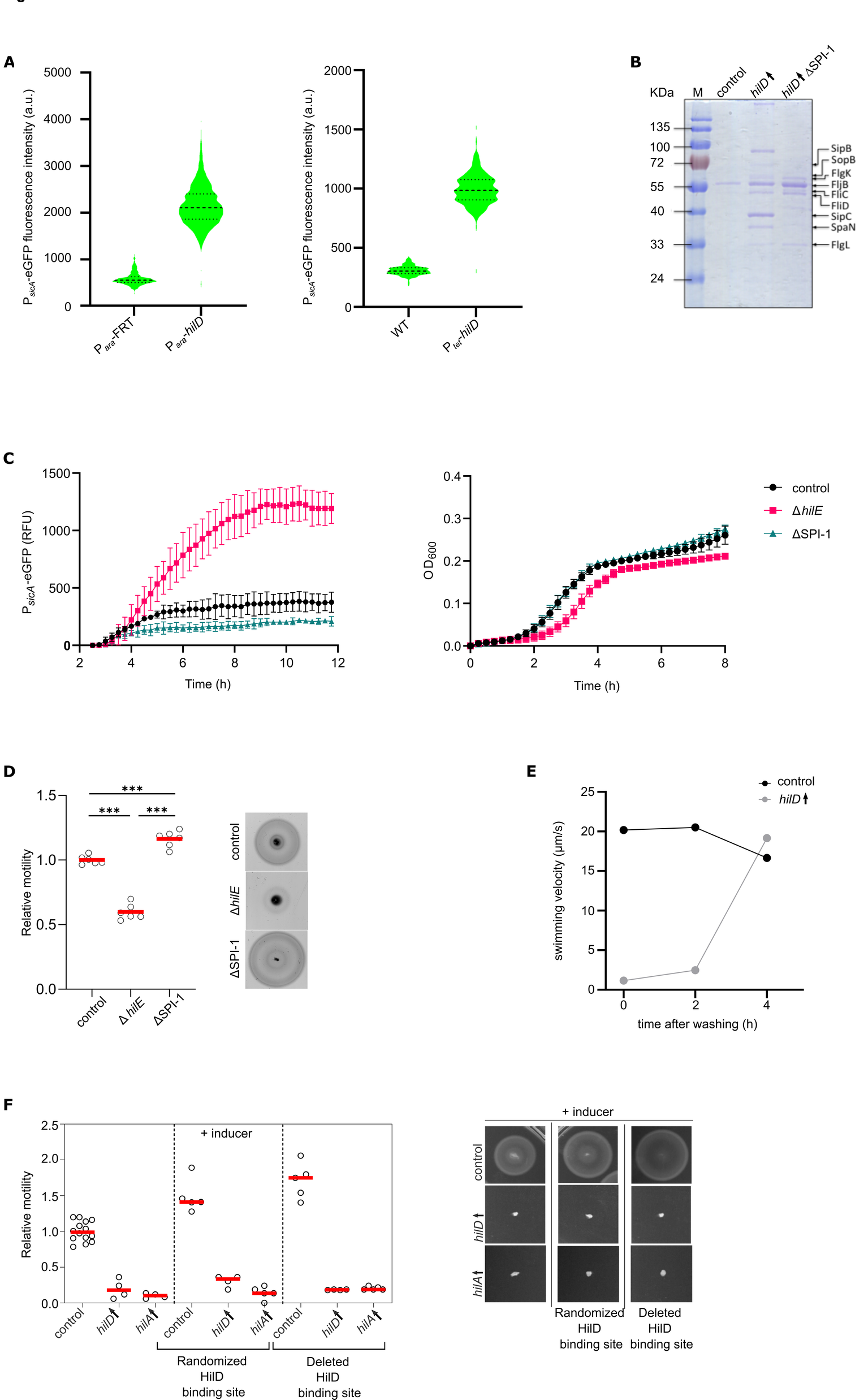

Supplement: S1 Fig — (A) Fluorescence intensities of a PsicA-eGFP fusion were measured in single cells as a reporter for hilD expression levels after inducing its expression from araBAD locus (Para) (left) and from the native locus under a tetracycline inducible promoter (Ptet) (right) using arabinose 0.2% and AnTc 100 ng/ml, respectively. Violin plots represent single-cell data from at least 200 analysed cells from one representative experiment. Strains analysed were EM899 (Para-hilD), EM900 (Para-FRT), EM228 (WT) and EM12302 (Ptet-hilD). (B) SPI-1 effector protein secretion into the culture supernatant after HilD induction using arabinose 0.2% was analysed via SDS-PAGE and Coomassie Blue staining. Results from one representative experiment are shown. Strains analysed were EM808 (control), TH16339 (hilD↑) and EM93 (hilD↑ ΔSPI-1). (C) Induction of SPI-1 gene expression in LB-Miller (1% NaCl) upon hilE deletion was monitored using a PsicA-eGFP transcriptional reporter fusion (left). Fluorescence intensities of PsicA-eGFP fusion. Fluorescence intensities of the cultures were measured in a microplate reader and normalised to the measured OD600 to give relative fluorescence units (RFU). The data points represent the calculated mean of twelve biological replicates. Error bars represent standard deviation. Strains analysed were EM228 (control), EM12232 (ΔhilE) and EM15052 (ΔSPI-1). Growth rates determined as a function of optical density at 600 nm (OD600) under the same conditions are shown to the right. The data points represent the calculated mean of six biological replicates. Error bars represent standard deviation. Strains analysed were TH437 (control), EM12177 (ΔhilE) and TH16265 (ΔSPI-1). (D) Swimming motility in soft-agar swim plates containing 1% NaCl was monitored at 37°C for 3.5 h in the following strains: TH437 (control), EM12177 (ΔhilE) and TH16265 (ΔSPI-1). Diameters of swimming halos were measured and normalized to the control (left). Representative swimming halos are shown (rig [file ppat.1011451.s001.tif]

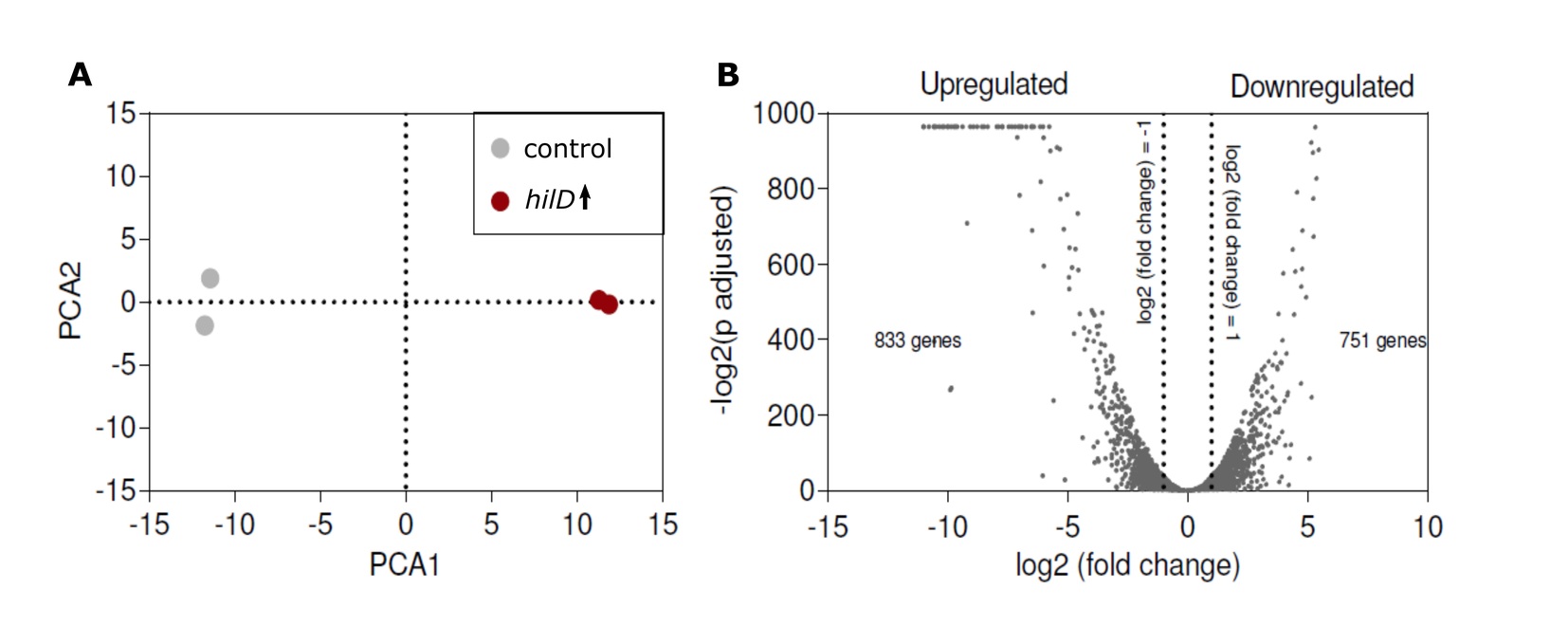

Supplement: S2 Fig — (A) PCA plot for the control (EM808) and HilD-induced strains (TH16339) in the presence of arabinose as an inducer for HilD overproduction. Within-sample normalisation was done based on the Transcripts Per Million (TPM). The TPM values of genes less than one variance were removed. The log2 of the TPM values were used to make the PCA plot. (B) Volcano plot showing all differentially expressed genes. DESeq2 was used to calculate the log2(fold change) and the corresponding adjusted p-values for the genes, by comparing the expression profile of control samples against HilD-induced samples. The results in (A) and (B) are calculated from two biological replicates. hilD↑: strain expressing hilD under an inducible promoter. (TIF) [file ppat.1011451.s002.tif]

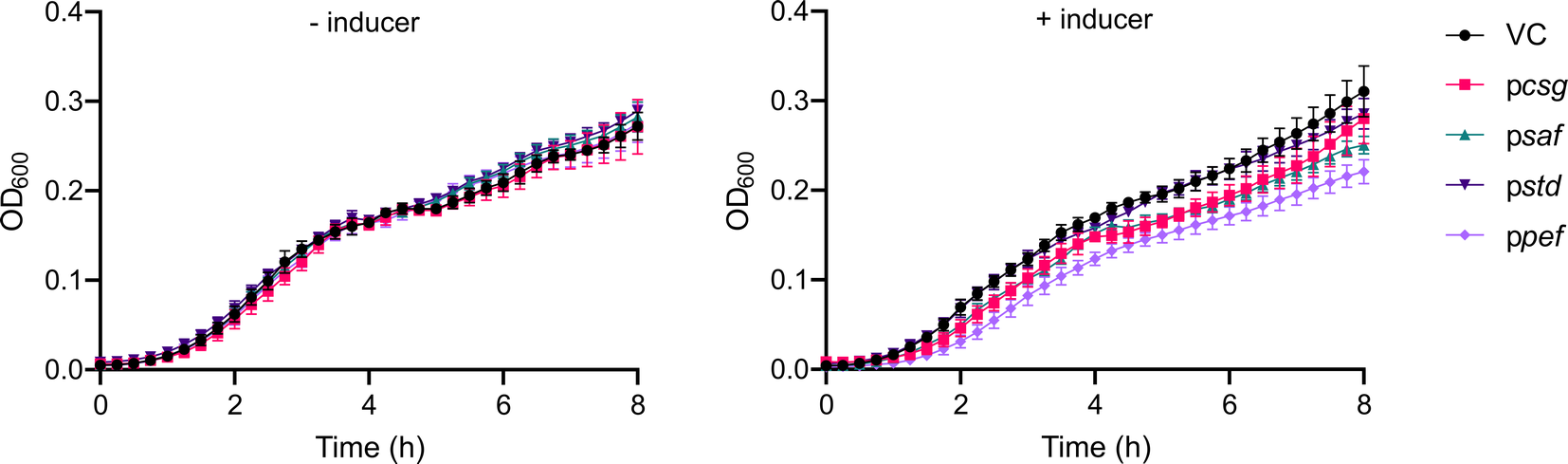

Supplement: S3 Fig — Growth rates were determined as a function of optical density at 600 nm (OD600) measured in a plate reader in absence (left) and presence (right) of 100 ng/ml AnTc as an inducer. The data points represent the calculated mean of six biological replicates at each time point. Error bars represent standard deviation. Strains analysed were EM12144 (VC, vector control), EM12145 (pcsg), EM12146 (psaf), EM12147 (pstd), EM12148 (ppef). AnTc: anhydrotetracycline. (TIF) [file ppat.1011451.s003.tif]

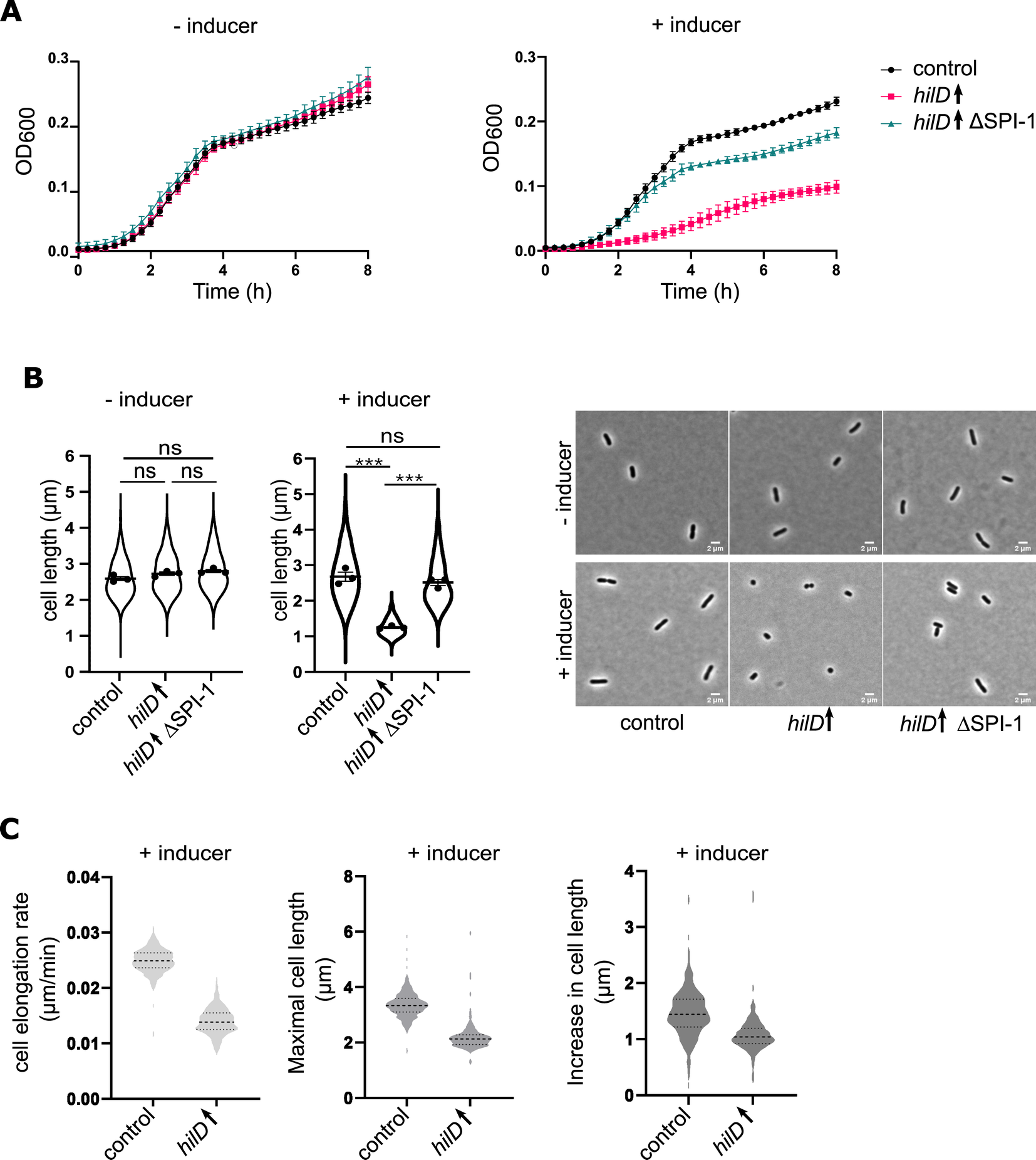

Supplement: S4 Fig — (A) Growth rates determined as a function of optical density at 600 nm (OD600) in absence and presence of AnTc for inducing HilD production. Individual points represent means of six biological replicates at each time point. Error bars represent the standard deviation. hilD↑: strain expressing hilD under an inducible promoter. AnTc: anhydrotetracycline. Strains analysed were TH437 (control), TH17114 (hilD↑), EM12479 (hilD↑ ΔSPI-1). (B) HilD induction results in decreased cell length (left) and coccoid morphology (right). Cell length (μm) of individual bacteria was determined by phase-contrast microscopy. Individual data points represent the averages of the single-cell lengths of independent experiments. Violin plots represent all single cell data values from a total of at least 400 analysed single cells. Horizontal bars (bold) represent the calculated average of three independent experiments. The error bars represent the standard error of mean and statistical significances were determined using a two-tailed Student’s t-test (***, P < 0.001; ns, P > 0.05). Representative images are shown in the right panel. the same strains as in (A) were analysed. (C) Time-lapse microscopy analysis reveals a decreased cell elongation rate (left), a decrease in the maximal cell length (middle) as well as decreased length elongation during a division cycle (right) upon HilD induction. Violin plots represent data values from at least 300 analysed cell lineages from a representative experiment. Strains analysed were EM12802 (control) and EM12803 (hilD↑). (TIF) [file ppat.1011451.s004.tif]

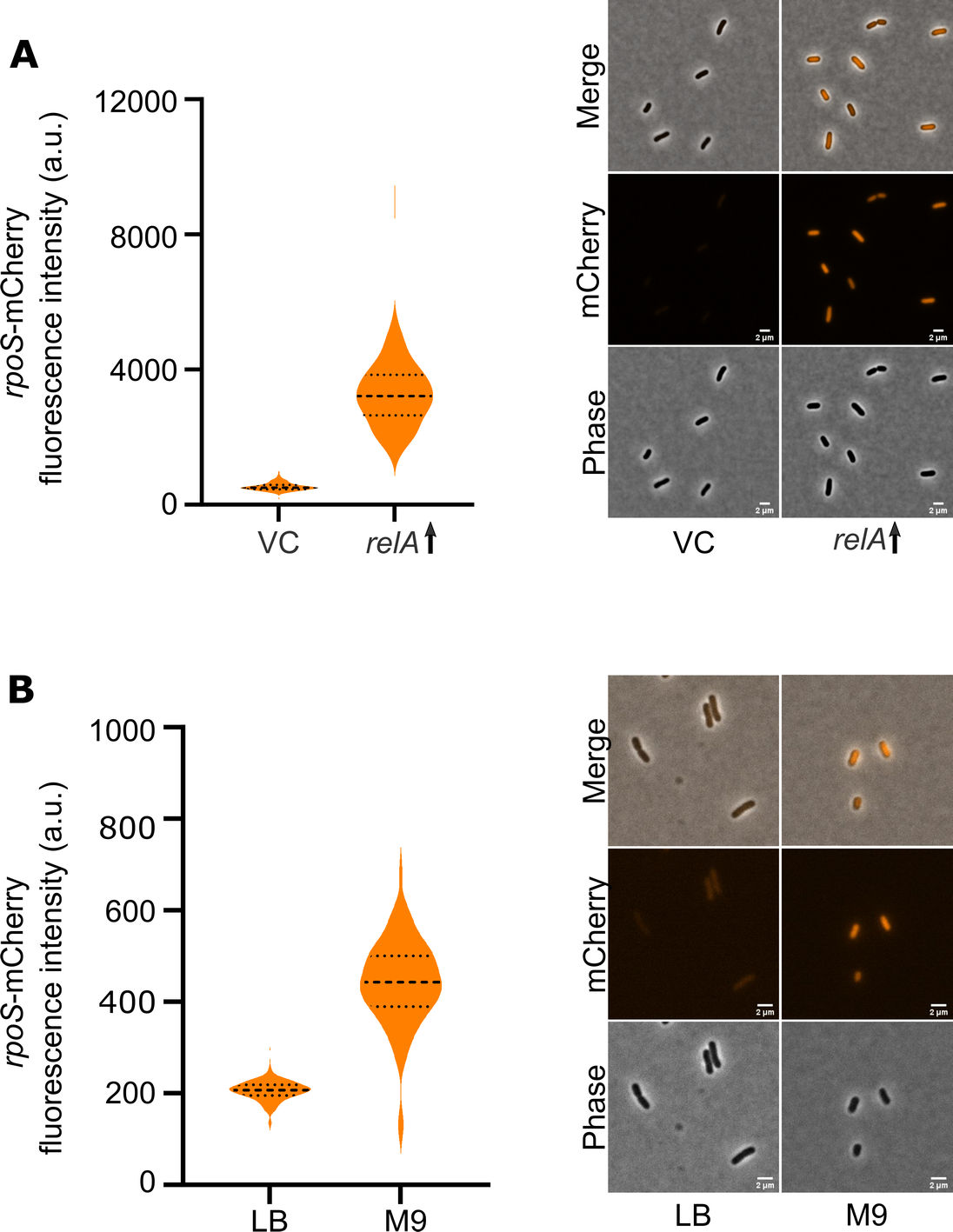

Supplement: S5 Fig — (A) Fluorescence intensities of rpoS-mCherry C-terminal translation fusions were measured in a strain overexpressing a constitutively active RelA mutant comprised of the first N-terminal 455 amino acids (relA↑). Fluorescence intensities in single cells were quantified using fluorescence microscopy (left). Violin plots represent data values from at least 200 analysed single cells from a representative experiment. Representative images are shown (right). Strains analysed were EM13226 (VC) and EM13227 (relA↑). (B) Assessment of rpoS-mCherry response to nutrient limitation in M9 minimal medium. Fluorescence intensities in single cells were quantified using fluorescence microscopy (left). Violin plots represent data values from at least 200 analysed single cells from a representative experiment. Representative images are shown (right). Scale bar is 2 μm. Strain analysed was EM13017. a.u.: arbitrary units. (TIF) [file ppat.1011451.s005.tif]

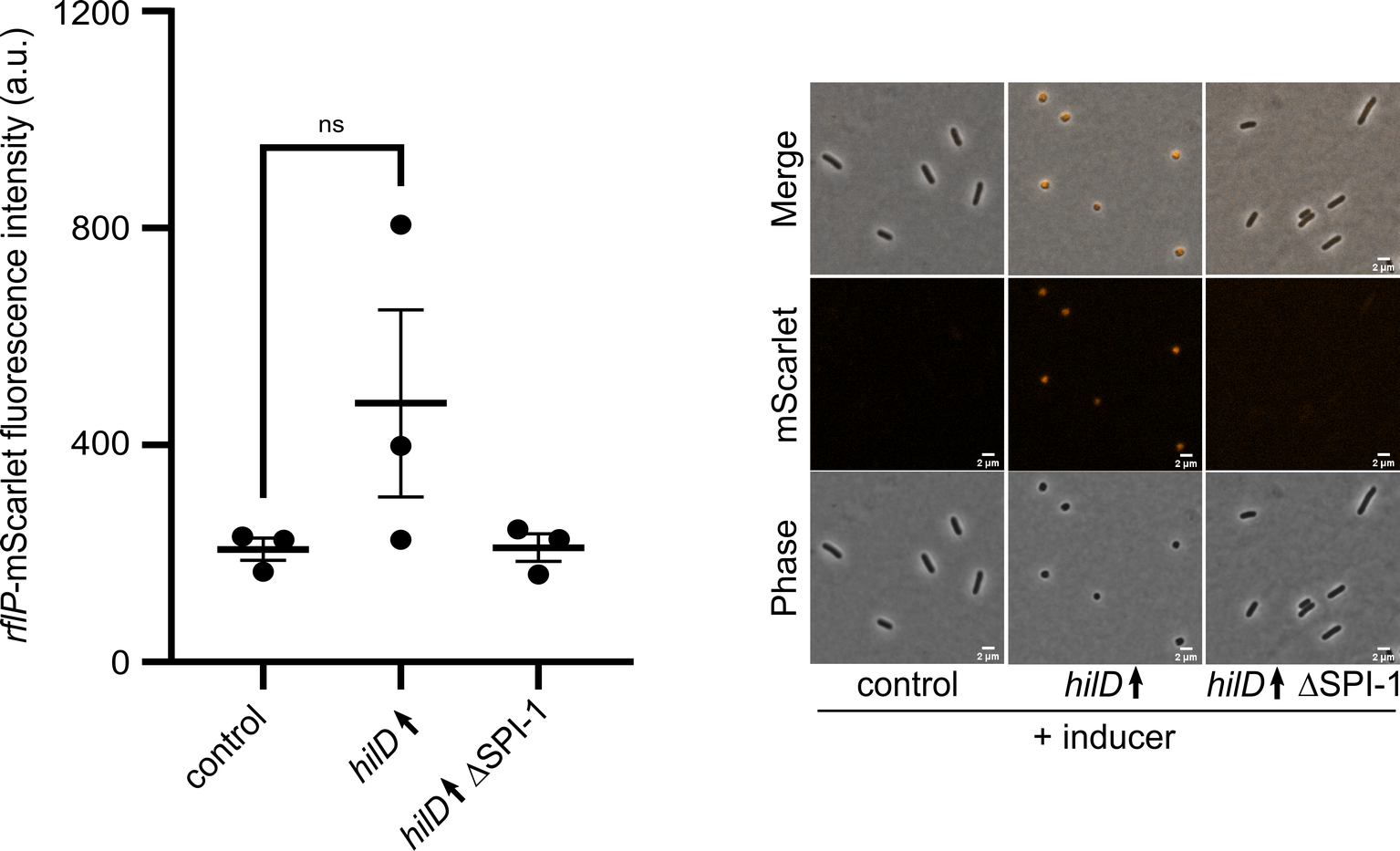

Supplement: S6 Fig — Strains were grown in presence of AnTc to induce HilD overproduction. Fluorescence intensities of rflP-mScarlet translational fusion were quantified in single-cells (left). Representative microscopy images are shown (right). Scale bar is 2 μm. Individual data points represent the averages of the single-cell data of independent experiments. Violin plots represent data values from at least 700 analysed single cells. Horizontal bars (bold) represent the calculated average of three independent experiments. Error bars represent standard error of the mean. Statistical significances were determined using a two-tailed Student’s t-test (ns, P > 0.05). Strains analysed were EM13097 (control), EM13278 (hilD↑) and EM13363. (hilD↑ ΔSPI-1). hilD↑: strain expressing hilD under an inducible promoter. AnTc: anhydrotetracycline. a.u.: arbitrary units. (TIF) [file ppat.1011451.s006.tif]

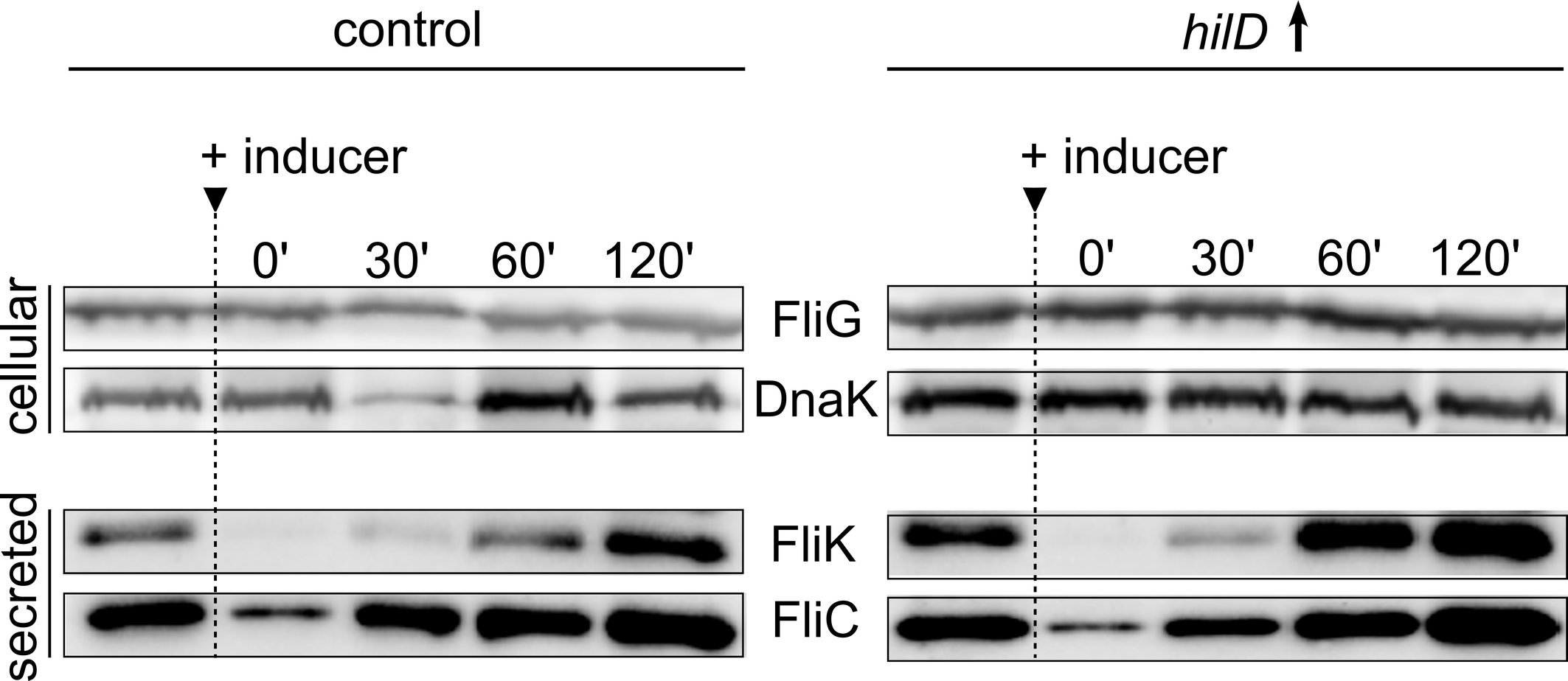

Supplement: S7 Fig — Strains were grown under HilD inducing conditions using AnTc followed by treatment with CCCP. Cells were then stained with the membrane potential sensitive dye DiSC3(5) and single-cell fluorescence intensities were quantified (left). Violin plots represent data values from at least 100 analysed single cells from a representative experiment. Representative microscopy images are shown (right). Scale bar is 2 μm. Strains analysed were TH437 (control), TH17114 (hilD↑) and EM12479. (hilD↑ ΔSPI-1). hilD↑: strain expressing hilD under an inducible promoter. AnTc: anhydrotetracycline. a.u.: arbitrary units. (TIF) [file ppat.1011451.s007.tif]

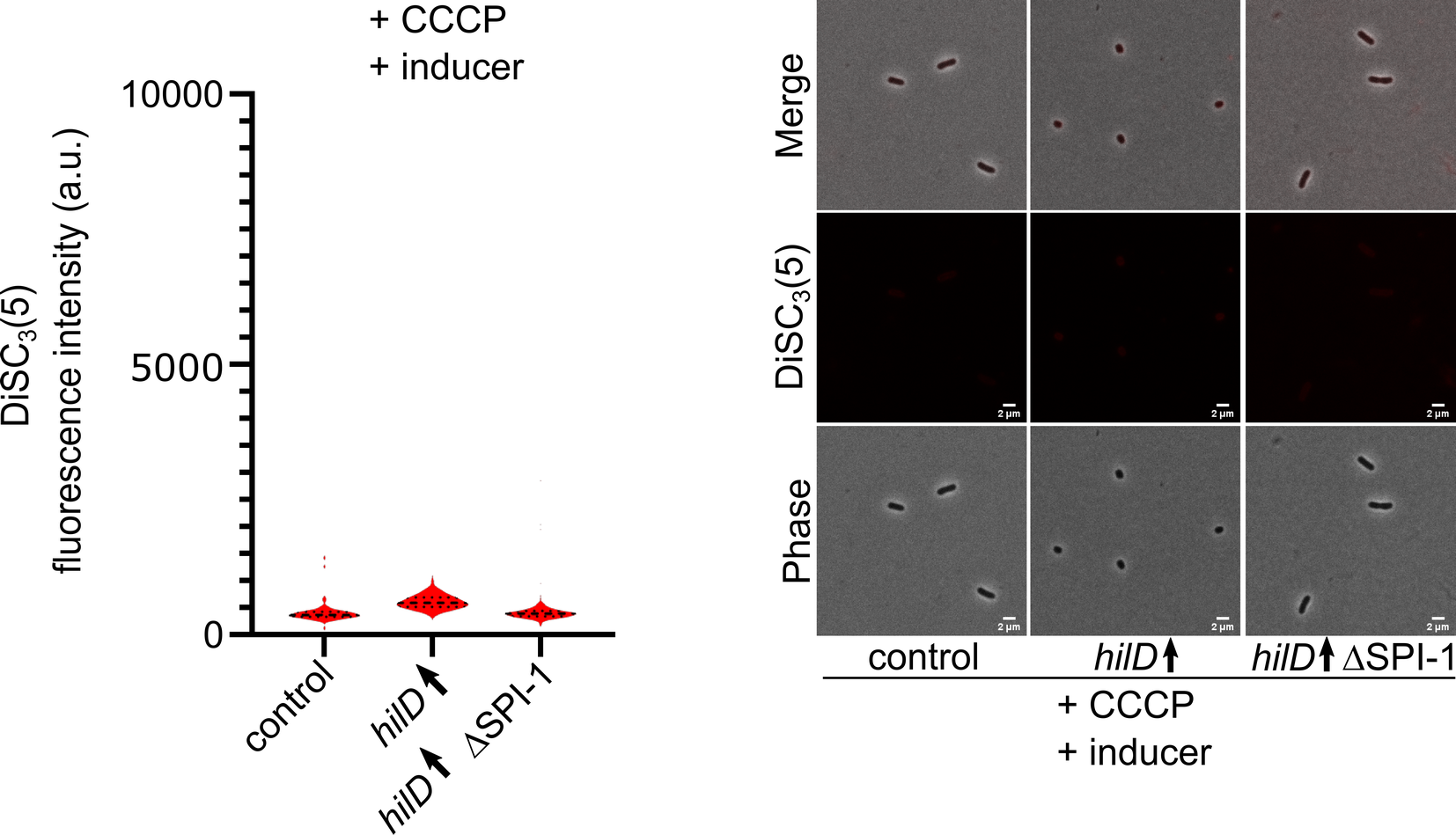

Supplement: S8 Fig — The levels of FliG, FliK and FliC were determined using western blotting in either the cellular fraction or the supernatant of the bacterial cultures as indicated. DnaK was used as a loading control. Samples were collected before HilD-induction and at the indicated time points after washing and inducing HilD by the addition of 0.2% arabinose. Results from a representative experiment are shown. Strains analysed were EM900 (control) and EM899 (hilD↑). (TIF) [file ppat.1011451.s008.tif]
