# Supplementary material for: SPI-1 virulence gene expression modulates motility of Salmonella Typhimurium in a proton motive force- and adhesins-dependent manner
Source: PLoS Pathog. 2023 Jun 14;19(6):e1011451. doi: 10.1371/journal.ppat.1011451 (PMC10298799; doi:10.1371/journal.ppat.1011451)
Supplement: S1 Table — (DOCX) [file ppat.1011451.s009.docx]

# Table S1. *Salmonella enterica* serovar Typhimurium strains used in this study.

| **Strain** | **Relevant characteristics** | **Reference** |
| --- | --- | --- |
| TH437 | *S. enterica* serovar Typhimurium wild-type strain LT2 | John Roth |
| TH16265 | Δ*invH*-*sprB*::FRT (deletes SPI-1) | This study |
| TH16339 | Δ*araBAD*1065::*hilD*^+^ | [1] |
| TH17114 | P*_hilD_*::*tetRA* (tetracycline inducible *hilD*) | This study |
| EM93 | Δ*araBAD*1065::*hilD*^+^ Δ*invH*-*sprB*::FRT | This study |
| EM228 | Δ*hutI-H*::P*_sicA_*-eGFP | This study |
| EM808 | Δ*araBAD*1005::FRT | [2] |
| EM831 | Δ*araBAD*1182::*hilD*_ΔHTH_ | This study |
| EM840 | Δ*sseA-ssaU*::FRT (ΔSPI-2) Δ*araBAD*1065::*hilD*^+^ | This study |
| EM899 | Δ*hutI-H*::P*_sicA_*-eGFP Δ*araBAD*1065::*hilD*^+^ | This study |
| EM900 | Δ*hutI-H*::P*_sicA_*-eGFP Δ*araBAD*1005::FRT | This study |
| EM930 | Δ*araBAD*1183::*hilA*^+^ | This study |
| EM3050 | P*_flhDC_*22343 (-598 to -554 A=C, T=G) (randomized HilD binding site) Δ*araBAD*1005::FRT | This study |
| EM3051 | P*_flhDC_*22343 (-598 to -554 A=C, T=G) (randomized HilD binding site) Δ*araBAD*1065::*hilD*^+^ | This study |
| EM3052 | P*_flhDC_*22343 (-598 to -554 A=C, T=G) (randomized HilD binding site) Δ*araBAD*1183::*hilA*^+^ | This study |
| EM3059 | ΔP*_flhDC_*(-598 to -554) (ΔHilD binding site) Δ*araBAD*1005::FRT | This study |
| EM3060 | ΔP*_flhDC_*(-598 to -554) (ΔHilD binding site) Δ*araBAD*1065::*hilD*^+^ | This study |
| EM3061 | ΔP*_flhDC_*(-598 to -554) (ΔHilD binding site) Δ*araBAD*1183::*hilA*^+^ | This study |
| EM12302 | Δ*hutI-H*::P*_sicA_*-eGFP P*_hilD_*::*tetRA* | This study |
| EM12144 | LT2 / pWSK29 (ApR) | This study |
| EM12145 | LT2 / p4830 (pWSK29-*tetR* P*_tetA_*::*csgBACEFG*, ApR) | This study |
| EM12146 | LT2 / p4393 (pWSK29-*tetR* P*_tetA_*::*safABCD*, ApR) | This study |
| EM12147 | LT2 / p4394 (pWSK29-*tetR* P*_tetA_*::*stdABCD*, ApR) | This study |
| EM12148 | LT2 / p4396 (pWSK29-*tetR* P*_tetA_*::*pefACDEF*, ApR) | This study |
| EM12177 | Δ*hilE* | This study |
| EM12232 | Δ*hutI-H*::P*_sicA_*-eGFP Δ*hilE* | This study |
| EM12354 | P*_hilD_*::*tetRA* Δ*siiABCDEF* (ΔSPI-4) | This study |
| EM12648 | SR11 Δ12 Δ*csgA*::FKF P*_hilD_*::*tetRA* | This study |
| EM12802 | LT2 / pBSB268 (pBAD18-GFP, ApR) | This study |
| EM12803 | P*_hilD_*::*tetRA* / pBSB268 (pBAD18-GFP, ApR) | This study |
| EM13065 | *rpoS*-SAGASA-mCherry (C-ter translational fusion) P*_hilD_*::*tetRA* | This study |
| EM13097 | *fliN*23482-mVenusNB-SAGASA (after aaM1) *rflP*23517::mScarlet (after aaM1) | This study |
| EM13100 | Δ*invH-sprB*::FRT *attP22*::[P*_hilD_*::*tetRA*] Δ*sseA-ssaU*::FRT (deletes SPI-2) | This study |
| EM13146 | *attP22*::[P*_hilD_*::*tetRA*] Δ*invH-sprB*::FCF (deletes SPI-1) | This study |
| EM13226 | *rpoS*-SAGASA-mCherry (C-ter translational fusion) / pTrc99a-FF4 (ApR) | This study |
| EM13227 | *rpoS*-SAGASA-mCherry (C-ter translational fusion) / pEM13227 (pTrc99a-FF4-*relA*(aa1-455), ApR) | This study |
| EM13276 | *attP22*::[P*_hilD_*::*tetRA*] Δ*invH-sprB*::FRT (deletes SPI-1) *rpoS*-SAGASA-mCherry (C-ter translational fusion) | This study |
| EM13278 | *fliN*23482-mVenusNB-SAGASA (after aaM1) *rflP*23518-mScarlet (before STOP) P*_hilD_*::*tetRA* | This study |
| EM13363 | *fliN*23482-mVenusNB-SAGASA (after aaM1) *rflP*23518-mScarlet (before STOP) *attP22*::[P*_hilD_*::*tetRA*] Δ*invH-sprB*::FKF (deletes SPI-1) | This study |
| EM15052 | Δ*hutI-H*::P*_sicA_*-eGFP Δ*invH*-*sprB*::FRT (deletes SPI-1) | This study |

# References

1. Singer HM, Kühne C, Deditius JA, Hughes KT, Erhardt M. The *Salmonella* Spi1 Virulence Regulatory Protein HilD Directly Activates Transcription of the Flagellar Master Operon *flhDC*. Journal of Bacteriology. 2014 Apr 1;196(7):1448–57.

2. Paradis G, Chevance FFV, Liou W, Renault TT, Hughes KT, Rainville S, et al. Variability in bacterial flagella re-growth patterns after breakage. Sci Rep. 2017 Apr 28;7(1):1282.
