# Supplementary material for: SPI-1 virulence gene expression modulates motility of Salmonella Typhimurium in a proton motive force- and adhesins-dependent manner
Source: PLoS Pathog. 2023 Jun 14;19(6):e1011451. doi: 10.1371/journal.ppat.1011451 (PMC10298799; doi:10.1371/journal.ppat.1011451)
Supplement: S2 Table — (DOCX) [file ppat.1011451.s010.docx]

# Table S2. Plasmids used in this study.

| **Plasmids** | **Relevant characteristics** | **Reference** |
| --- | --- | --- |
| pWSK29 | low copy number cloning vector (ApR) | Michael Hensel |
| p4830 | pWSK29-*tetR* P*_tetA_*::*csgBACEFG* (ApR) | [1] |
| p4393 | pWSK29-tetR P*_tetA_*::*safABCD* (ApR) | [1] |
| p4394 | p4394 (pWSK29-tetR P*_tetA_*::*stdABCD* (ApR) | [1] |
| p4396 | p4396 (pWSK29-tetR P*_tetA_*::*pefACDEF* (ApR) | [1] |
| pBSB268 | pBAD18-GFP (ApR) | [2] |
| pTrc99a-FF4 | IPTG-inducible expression vector (ApR) | [3] |
| pEM13227 | pTrc99a-FF4-*relA*(aa1-455) (ApR) | This study |

# References

1. Hansmeier N, Miskiewicz K, Elpers L, Liss V, Hensel M, Sterzenbach T. Functional expression of the entire adhesiome of *Salmonella enterica* serotype Typhimurium. Sci Rep. 2017 Dec;7(1):10326.

2. Dongre M, Singh B, Aung KM, Larsson P, Miftakhova R, Persson K, et al. Flagella-mediated secretion of a novel *Vibrio cholerae* cytotoxin affecting both vertebrate and invertebrate hosts. Commun Biol. 2018 Jun 7;1(1):1–12.

3. Ohnishi K, Fan F, Schoenhals GJ, Kihara M, Macnab RM. The FliO, FliP, FliQ, and FliR proteins of *Salmonella* typhimurium: putative components for flagellar assembly. Journal of Bacteriology. 1997 Oct;179(19):6092–9.
