# Supplementary material for: SPI-1 virulence gene expression modulates motility of Salmonella Typhimurium in a proton motive force- and adhesins-dependent manner
Source: PLoS Pathog. 2023 Jun 14;19(6):e1011451. doi: 10.1371/journal.ppat.1011451 (PMC10298799; doi:10.1371/journal.ppat.1011451)
Supplement: S3 Table — (DOCX) [file ppat.1011451.s011.docx]

# Table S3. Primers used in this study.

| **Number** | **Name** | **Sequence** |
| --- | --- | --- |
| 9 | Ara117fwd | CCACATTGAATATTTGCACAGCG |
| 59 | 5'-ParaB-hilA_50C-fw | actgtttctccatacctgtttttctggatggagtaagacgATGCCACATTTTAATCCTGT |
| 60 | 3'-ParaD-hilA_49C-rv | tcatcaacgcgccccccatgggacgcgtttttagaggcaTTACCGTAATTTAATCAAGCG |
| 128 | 5'-Dhut-PsicA-FPs-fw | aatgtctatacaacccatgacaggcaagagagcgacaggaGCCGCGTAAGGCAGTAGCGA |
| 129 | 3'-Dhut-PsicA-eGFP-rv | tgggcaccaccccggtgaacagttcttcgcctttgctcatTACTTACTCCTGTTATCTGT |
| 481 | 3'-hilD-dHTH_50C-fw | atcaacgcgccccccatgggacgcgtttttagaggcattATGACGAAGATATAATGTTGT |
| 5043 | 5’-DhilE-KanSceI_fw | ttgtcagcctgctttgccgcatgataatcacggaggggggAGGGTTTTCCCAGTCACGAC |
| 5044 | 3’-DhilE-KanSceI_rv | acagcatcgcccactgcgagtccgcaagcttgttttgtccTGCTTCCGGCTCGTATGTTG |
| 5045 | 5’-DhilE-clean-del_fw | GTGGAATTAAGTAATTTCTC |
| 5046 | 3’-DhilE-clean-del_rv | acagcatcgcccactgcgagtccgcaagcttgttttgtccCCCCCCTCCGTGATTATCAT |
| 5049 | 5’-Dspi-4-KanSceI_fw | acaaaaacattttattcacaatgtaatatcaggagacaacAGGGTTTTCCCAGTCACGAC |
| 5097 | DsiiA-F-KanSceI_new_rv | aagcagtaccacctgataacagcgacaagcgctgcttattTGCTTCCGGCTCGTATGTTG |
| 5098 | DsiiA-F_clean-del_new_fw | AATACGTATGGTTATAACGC |
| 5099 | DsiiA-F_clean-del_new rv | aagcagtaccacctgataacagcgacaagcgctgcttattGTTGTCTCCTGATATTACAT |
| 5340 | 5’-attP-Ptet-hilD_fw | aatgcgaaggtcgtaggttcgactcctattatcggcaccaGGGATTCCTGATGAAAATAG |
| 5341 | 3’-attP-Ptet-hilD_rv | tttttgagaaatgaggttgtacataagtgattgatttagaGTTAATGCGCAGTCTGAATT |
| 5591 | 3’-XbaI-relA (aa1-455)_rv | ctagtctagatcattattaCAACTGATAGGTGAATGGCA |
| 5718 | 5’_SAGASA-mCherry_fw | agcgcgggtgctagcgcgGTTTCCAAGGGCGAGGAGGA |
| 5719 | 3’_mCherry_rv | TTATTATTTGTACAGCTCAT |
| 5720 | 5’_rpoS-SAGASA-mCh_fw | gcagacgcaggggctgaatatcgaagcgctgttccgcgagAGCGCGGGTGCTAGCGCGGT |
| 5721 | 3’_rpoS- mCh_rv | gccagtcgacagactggcctttttttgacaagggtacttaTTATTATTTGTACAGCTCAT |
| 5748 | 5'-NdeI-relA_fw | cgccatatgGTCGCGGTAAGAAGTGCACA |
| 5751 | 5’_rpoS-tetRA-beforestop_fw | gcagacgcaggggctgaatatcgaagcgctgttccgcgagTTAAGACCCACTTTCACATT |
| 5752 | 3’_rpoS-tetRA-beforestop_rv | gccagtcgacagactggcctttttttgacaagggtacttaCTAAGCACTTGTCTCCTG |
