# Supplementary material for: SPI-1 virulence gene expression modulates motility of Salmonella Typhimurium in a proton motive force- and adhesins-dependent manner
Source: PLoS Pathog. 2023 Jun 14;19(6):e1011451. doi: 10.1371/journal.ppat.1011451 (PMC10298799; doi:10.1371/journal.ppat.1011451)
Supplement: Attachment — Submitted filename: Response to reviewers.docx [file ppat.1011451.s013.docx]

# We would like to thank the three reviewers for their expertise and their rigorous reading of our manuscript. We are thankful for their feedback and suggestions and for their constructive criticism that helped us to improve our manuscript.

# We are confident that we were able to address all the comments. Changes in the revised manuscript are highlighted in blue to facilitate reading and our point-to-point responses to the reviewer’s comments are provided below.

# Reviewer #1

# The manuscript by Saleh et al explores the effects of HilD activation on motility, stringent response and adhesin production in Salmonella Typhimurium. Specifically, they show that despite its role in activation of flhD expression, HilD overexpression reduces motility and increases the expression of several previously described adhesin gene clusters. The authors further tie this phenotype to SPI-1-dependent induction of the stringent response and decrease of PMF. The experiments are elegant, the data is convincing and the setups are well-described. Furthermore, the work touches upon an interesting topic of how certain transcriptional programs in S. Typhimurium can dissipate the cellular energy pool, ultimately leading to phenotypic changes. I have only spotted a few points that the authors might want to consider prior to publication.

# RE: We appreciate your comprehensive positive evaluation and insightful comments. We have taken your suggestions into account and conducted several additional experiments. As a result, we have incorporated growth rates, P*_sicA_*-eGFP expression and motility phenotypes in high salt media in different genetic backgrounds to strengthen our manuscript. The results are shown in Supplementary Figures S1C-D.

# Major comments:

1. **Although overexpression of HilD results in a set of well described and convincing phenotypes, I wonder if the authors may want to discuss how these phenotypes might change, if experiments had been performed under more physiologically relevant conditions. Using LB with 0.3M NaCl might be one option. It should be discussed if that accumulation of HilD and concomitant overexpression of type 3 secretion systems in the presented experiments might result not only affect the normal hilD regulon, but also yield some unspecific response phenotypes (Fig. S2B, differential expression of ca 1600 genes doesn’t look hilD-specific anymore). Could this be approached by comparing the published transcriptome data of hilD mutants vs wt (Colgan et al) side by side with the new data on inducible hilD expression?**

RE: Thank you for the suggestion. We tested the motility and growth phenotypes in WT strain, a strain deleted for *hilE*; a repressor of HilD as well as a strain deleted for SPI-1. The experiments were performed in LB with 1 % NaCl. We observed a motility defect in the Δ*hilE* accompanied with a decrease in growth rate compared to the control. The deletion of SPI-1 restored both motility and growth phenotypes. *The results are now presented in Figure S1C-D.*

We agree that the longer periods of HilD-induction might lead to differential expression of genes that are not under direct control of HilD, in contrast to the data published by Colgan et al and Smith et al. This observation aligns with the fact that HilD-induced cells appear to be undergoing a stringent response, which likely results in a global effect on the transcriptome. Importantly, we confirmed the differential regulation of selected genes, which encode adhesins, by RT-qPCR and, the induction of HilD resulted in increased adhesion to cultured epithelial cells.

2. **The authors cite literature showing the roles of PMF not only in flagellar rotation, but also in flagellar synthesis (lines 334-335). They additionally link the induction of stringent response upon HilD overexpression to PMF dissipation (Figure 6). However, I am not sure why the flagellation level of such cells remained largely unaffected (Figure 5). Can the authors comment on that?**

RE: Thank you for your comment. We acknowledge that the results of the flagella counts did not align with our initial expectations. We hypothesized that a stringent response would likely activate RflP, resulting in predominantly non-flagellated cells. Although we did observe a slight increase in RflP levels, the differences were not statistically significant. We also noted a minor decrease in the average number of flagella per cell in the HilD-induced cells. However, when accounting for differences in cell length, the number of flagella per unit cell length remained consistent.

We speculate that the upregulation of FlhDC might force the re-allocation of cellular resources, which are relatively scarce due to the HilD-mediated stringent response, towards flagella synthesis. This is considering that the cells continue to actively translate proteins, albeit at a reduced rate, at least during the time periods used in our experiments. The described role of flagella in facilitating the adherence and invasion of epithelial cells (PMID: 32886602, PMID: 32332720) might explain why *Salmonella* would invest the scarce cellular resources for flagella synthesis. *We have now included a discussion of this point in lines 485-489 of the revised manuscript.*

3. **Figure 7: the model proposed by the authors relies on the assumption that HilD is only activated (resulting in repressed motility) upon the contact between STm and epithelial cells. However, it has been previously shown that HilD is activated by several environmental cues in the gut lumen (Ellermeier et al, 2008; Golubeva et al, 2012; Diard et al, 2013). I would say that the lines 419-430 are indeed highly speculative and not backed up by corresponding data. The authors may want to modify this statement.**

RE: Thank you for your valuable feedback. We concur that the activation of HilD does not necessitate contact with epithelial cells. However, it's worth noting that the secretion of effectors is significantly activated upon cell contact (PMID: 16406778, PMID: 18478101, PMID: 16107539). Moreover, it has been suggested that additional injectisomes are assembled upon host cell contact, facilitating prompt secretion of the formed effectors pool (PMID: 25524451). These events could lead to PMF depletion. *We have now elaborated on this in lines 496-504 of the revised manuscript.*

We further acknowledge that future work is needed to fully investigate all aspects of our proposed model. We have improved our discussion regarding the observed flagellation and the kinetics of the injectisome activation and included relevant literature. We hope these additions will provide a clearer understanding of how our data aligns with the proposed model. *The background information and the proposed model are now detailed in lines 504-517. We have also made corresponding modifications to Figure 7.*

# Minor comments:

1. **Line 268: It would be interesting to compare the growth rate and cell length phenotypes of hilD expression to the published data from Arnoldini et al 2014 and Hockenberry et al. 2021.**

RE: Thanks for suggesting those references. Generally speaking, the phenotypes align, although there are some discrepancies in the quantification results, likely due to differences in the experimental setups used. *We now discuss these references in greater detail in lines 444-455 of the manuscript.*

2. **Lines 312-320: Authors rule out the potential role of RflP in the observed motility phenotype. However, in contrast to the quantification data, the microscopy image suggests a difference. The authors may want to choose a more representative image?**

RE: We appreciate your observation. While we cannot completely rule out a contribution of RflP, it appears that it may not play a significant role, at least at the time points tested. This is because the bacteria remained flagellated, and the slight decrease observed in the number of flagella per cell does not correspond to the dramatic decrease in swimming speed. We did note an increase in RflP levels in all replicates upon HilD-induction, but it was statistically non-significant, likely due to high day-to-day variance.

**3. Line 56: no need for comma after “although”**

RE: Thank you. The text has been corrected.

**4. Line 149: “exclude”, not “exclude for”**

RE: Thank you. The text has been corrected.

**5. Statistical test results should be added in Fig. 1, Fig. 2BD, Fig. 3A**

RE: We have now included the results of statistical tests in Figures 1, 2BD, and 3A.

**6. Fig. 2DE. It would be interesting for the readers, if the authors could discuss the differences in the two motility phenotypes of pcsg, psaf and pstd.**

RE: Thanks for pointing this out. We believe that any phenotype observed in the soft-agar plates could potentially be attributed to differences in growth rates upon induction.

We hypothesize that the glass flow chambers used for the single-cell swimming experiment provided a suitable surface for the adhesins to adhere to, thereby abolishing motility. *We now elaborate on these differences in lines 217-226 and Figure S3.*

**7. Line 337-343: The authors may want to discuss that besides T3SS effector secretion, errors or normal steps of T3SS apparatus assembly may also dissipate the PMF.**

RE: Thank you for the suggestion. *We now discuss the effect of secretin mislocalization on PMF in lines 465-469.*

**8. References 65 and 73 appear to be partially deleted?**

RE: Thank you. The references were corrected.

# Reviewer #2

# Saleh et al. conclude that induction of the SPI1 T3SS leads to a loss of PMF and hence, loss of motility. Various adhesins are also induced. The overall data support the conclusion. This leads to an intriguing model in which the cell turns on SPI1 and stops swimming, potentially enhancing invasion. The issue is that this model is counter to the coordinate transcriptional induction of the two systems, a point that needs to be more fully discussed.

# RE: We sincerely thank you for your thoughtful review and positive feedback on our manuscript. As detailed in our response to point 11, we have enhanced our discussion to provide a more comprehensive explanation of our current working model.

# Major comments:

**None**

# Minor comments:

1. **Line 79. “A battery of DNA-binding proteins, such as … HilD, HilC, and RtsA…” While numerous regulatory systems affect expression of SPI1, most function indirectly. This sentence makes HilD seem like a minor player.**

RE: The sentence was rephrased accordingly.

2. **Line 82. A pet peeve. I think calling HilA and an “OmpR/ToxR-type transcriptional regulator” can be confusing. The uninitiated might think it is a two-component regulator. It is only the DNA binding domain that is similar to OmpR.**

RE: It is true that only the DNA binding domain is similar to OmpR and we have re-phrased the sentence.

**3. The regulatory interplay between flagella and SPI1 is obviously complicated. You could use a model slide to make it clear.**

RE: Thanks for the suggestion. A figure has been added to illustrate the complex regulatory interplay between flagella and SPI-1 (*Figure1A*).

**4. Line 137. The strain description is not clear. “…a chromosomal copy of hilD under the control of an inducible promoter, including an ectopic locus (araBAD) and the native locus.” Is the “native locus” a tet promoter? Clarify. You also should point out that the native locus is intact in the ara:hilD strain.**

RE: Thank you for pointing out the ambiguity. We have now rephrased the sentence for clarity.

**5. Line 145. You do not describe the strain ectopically expressing hilA.**

RE: We have added a descriptive sentence for the strain ectopically expressing *hilA*.

**6. Fig 2B. You need statistical analyses. Is any of this significant?**

RE: We have now included statistical significance in Figure 2B.

**7. Line 195. “Strains expressing hilD under the native promoter and deleted for SPI1…” This makes no sense. If SPI1 is deleted, so is hilD. This strain is ara::hilD, correct?**

RE: Yes, thank you. The sentence regarding the strains expressing *hilD* under the native promoter and deleted for SPI-1 has been corrected.

**8. Line 231. Deletion of SPI1 blocks the effect. In your strains, deletion of SPI1 is likely to significantly decrease the overall amount of HilD/HilC/RtsA in the cell, given the ectopic HilD expression will induce the native loci with autoinduction intact. Your data seem clear and induction of hilA also confers the phenotype. However, you should likely acknowledge this caveat.**

RE: Thank you for highlighting this point. *We now acknowledge this caveat in lines 265-268 of the manuscript.*

**9. Line 264. What is the evidence that “translation rate” per se is affected? The word “translation” is not used in Ref 53.**

RE: Thank you for your comment. We have revised the sentence regarding the effect on the "translation rate" for accuracy.

**10. Figure 6. How long were these cells induced? Does time matter?**

RE: The cells in Figure 6 were induced for the entire duration of the main culture, approximately 2.5 hours. However, as shown in Figure 1, we observed a sharp drop in velocity 45 minutes after induction of an exponentially growing culture.

**11. Although your model is intriguing, it goes against the apparent coordinate transcriptional induction of the two systems. Why would HilD turn on FlhDC if the goal is to stop swimming? You need to at least comment on this. I am wondering if the timing of these events matters and perhaps needs more careful explanation and consideration. The materials and methods implies that the cells in Fig 6 were incubated for 5 mins? Fig 1 suggests that loss of motility occurs after 45 mins?**

RE: We appreciate your insightful comments. While our model may seem counterintuitive, we believe it provides a plausible explanation for the observed phenotype. We have revised the discussion (*lines 485-489, 496-513*) to better explain our proposed model and the potential role of flagella in facilitating adherence to and invasion of host epithelial cells, rather than for motility.

The 5-minute period mentioned in the materials and methods refers to the incubation time for DiSC3(5) staining. As in most of our experiments, the bacterial cultures were first grown to mid-logarithmic phase under HilD-inducing conditions before performing DiSC3(5) staining.

# Reviewer #3

# In this manuscript, Saleh and colleagues describe the effect of overexpression of HilD, the master transcriptional activator of the Salmonella SPI-1 injectisome, on bacterial cell physiology, especially motility. This study follows up on an earlier study by the same group (Singer et al., J Bact 2014), where HilD overexpression led to increased transcription of flhDC, the master transcription activator of the flagellum. In apparent contrast, the current study shows a strong reduction of motility upon HilD overexpression, which depends on the presence of SPI-1. Overexpression of HilD in presence of SPI-1 also resulted in an activation of the stringent response and reduced membrane potential. The authors propose that these data indicate a strategy to quickly change the bacterial lifestyle from mobile to adhesive upon contact to host cells.

**The interplay of different molecular mechanisms (such as motility and protein secretion) is an important and so far poorly understood aspect of bacterial virulence. This manuscript addresses it with a wide range of experiments, which are presented in a clear and very attractive manner. Similarly, the proposed model is intuitive. However, the data mainly describe effects of overexpression of a known transcription factor, HilD, and confirm that these effects depend on the presence of its known main target, the SPI-1 injectisome. Whether and to which extent the results show a biologically relevant mechanism therefore remains largely unclear. Further, the reason for the – at least on the first look – clear discrepancy between the results of the 2014 paper and this manuscript is not revealed.**

# RE: Thank you for you for the constructive and positive assessment of our work and we are grateful for the time and effort put into the review.

# Major comments:

1. **Almost all experiments were performed with strains overexpressing HilD (usually in wild-type and SPI-1 deletion backgrounds). A direct comparison of wild type and SPI-1 deletion without the overexpression of HilD would often have been possible and more physiologically relevant. Although overexpression of a transcriptional activator can yield insights into its working mechanism, interpretation of physiological effects of native expression of the target genes are difficult. The fact that strong overexpression of HilD and in turn the SPI-1 injectisome (a large membrane-spanning complex), leads to effects including loss of membrane potential, does not necessarily allow conclusions about the native interaction of SPI-1 expression and motility, as claimed in the title and throughout the manuscript. These claims should therefore be corrected.**

RE: We appreciate your suggestion. In response, we have tested the motility and growth phenotypes in different genetic backgrounds in SPI-1 inducing media. The findings are described in more details in our response to reviewer #1 first major comment. *The results are presented in Figure S1C-D of the revised manuscript.*

2. **The reason for the apparent contradiction between the results of Singer et al. (overexpression of HilD leads to increased transcription of flhDC) and this manuscript is a critical question, but remains unclear. The manuscript almost actively steers clear from data that would allow to clarify this point. For example, the transcriptomics data shown in Figure 2A for “selected genes” only includes two flagellar genes. To evaluate the data and any differences between the two studies (and potential reasons), the complete dataset must be made available, which can be done using a preliminary protected link for the reviewers. In addition, at least the genes presented in Figure 2A plus all flagellar genes must be listed with their names and the actual data in a Supplementary Table. Furthermore, it would be very helpful to show the protein levels of at least some flagellar components.**

RE: Thank you. We believe that the data from the two studies are not contradictory. In the study by Singer et al., HilD was shown to activate the transcription of *flhDC* using qRT-PCR and transcriptional *lacZ* fusions. Moreover, the binding site for HilD was mapped to the P5 promoter region of *flhDC* using EMSA and DNA footprinting assays. In our study, we aimed to characterize the consequences of HilD binding and *flhDC* upregulation. We acknowledge that the observed motility phenotypes upon HilD-induction were unexpected. However, the *flhDC* transcript levels were indeed increased upon HilD-induction in our transcriptome data set and also in the data set published by Smith et al. (PMID: 27601571), consistent with the findings of Singer et al..

*We have updated Table S4 to include the names of the genes that were either up- or downregulated, whenever possible, in addition to the TPM values and the differential expression analysis of all the detected genes (listed by Gene ID).* The flagella genes *flhD* and *fljB* were among the upregulated genes in addition to the chemotaxis-related genes *cheB/Y/Z*, *mcpA* and *aer.* On the other hand, some flagellar genes were down-regulated including *fliE*, *fliF*/*J*/*K* and *flgF/G/I*.

As suggested, we assessed the levels of different flagella proteins using Western blotting. The results indicate that until 2 h after HilD-induction, the bacteria are still capable of producing and secreting flagellar proteins. *The results are presented in Figure S7 of the revised manuscript.*

3. **The abstract states that “overproduction of several adhesion systems phenocopied the HilD-induced motility defect”. While this is true for swimming velocities in a glass flow chamber (which, as the authors themselves state, is probably an artifact of attachment to the glass surface), this does not seem to be the case for the other observed phenotypes. Relatedly, the following sentence states that “combination of SPI-1-dependent depletion of the PMF and upregulation of adhesins (…) allows flagellated Salmonella to rapidly modulate their motility.” Given that deletion of SPI-1 alone rescues most phenotypes, it appears to be the main (or only) factor for them and the role of adhesins minor, if present at all.**

RE: We appreciate your feedback. We acknowledge that a subpopulation adheres to the glass surface even in the case of the WT. However, we believe that the total loss of motility across the entire population is unlikely to be solely due to this artifact, as also demonstrated in Figure 2E. We interpret the discrepancy between the motility phenotype in soft agar and in liquid medium by the fact that bacteria expressing adhesins may attach to the glass surface oft he flow chamber used in the single-cell setup, but not in the soft-agar plates. *In response to your feedback, we have addressed this concern in lines 220-226 of the revised manuscript.* The potential effect of adhesins is further supported by the enhanced adhesion of HilD-induced bacterial cells to cultured epithelial cells (Figure 2C). Overall, we concur that SPI-1 is the primary factor, and the role of adhesins is minor, but they may still play a role.

4. **Is it really that surprising to find that flagella and SPI-1 injectisomes are adversely regulated in Salmonella? Similar mutually negative regulation has been found in several other cases, including E. coli, Yersinia enterocolitica and Pseudomonas aeruginosa (****Iyoda et al, J Bact (2006);** **Bleves et al, J Bact (2002); Soscia et al. J Bact (2007)). These references should be discussed and the text adapted accordingly.**

RE: Thank you for your comment and for highlighting those previous studies. We found it somewhat surprising to observe such a drastic decrease in motility despite the upregulation of FlhDC (and other flagella genes) previously noted in several studies, including ours, upon HilD-induction. The studies you mentioned indeed report an adverse effect of flagella gene expression on the vT3SS and/or reciprocal regulation between the two systems. While such reciprocal regulation also exists in *Salmonella*, flagella have been demonstrated to be necessary in Salmonella for mediating adhesion and invasion of host cells (PMID: 32886602, PMID: 32332720), not just for motility. This might explain why *Salmonella* would maintain flagella even when there is no critical need for motility. *We have discussed these studies in detail in lines 474-489 of the revised manuscript.*

# Minor comments:

1. **The number of biological replicates should be indicated for each experiment.**

RE: We have now included the number of biological replicates in the figure captions.

2. **In the proposed model, would the reversibility of the commitment to attachment (e.g., in case of failed interactions with the host cell) be an advantage of the mechanism proposed in the authors’ model over the ejection of flagella?**

RE: We appreciate your insightful comment. Indeed, this aspect aligns well with our model, in addition to the reported role of flagella in facilitating successful interactions with host cells. *We now discuss the proposed advantage of maintaining flagellation, even in the absence of an apparent need for motility, in lines 504-513 of the revised manuscript.*

**3. Line 91, ref. 29 does not deal with injectisomes, please add suitable reference.**

RE: Thank you for pointing this out. To our knowledge, there is no reference that provides a model for the energy consumption associated with injectisome assembly and effector secretion similar to the one cited. However, we have added several references that discuss its dependence on PMF and ATP, and its homology in this aspect to the flagellar T3SS.

**4. Line 98, please describe how FliZ regulates HilD in ref. 34.**

RE: The relevant findings were added.

**5. Line 110, in contrast to the claim that “HilD activates the expression of SPI-2 genes”, ref. 43 explicitly states (in the abstract) that “HilD is not required for SPI-2 regulon expression under the in vitro growth conditions that are thought to resemble the intracellular environment”.**

RE: By this statement, the authors wanted to highlight that a medium with an acidic pH did not result in SPI-2 gene induction as expected. Additionally, in other minimal media they did not detect an effect for HilD on *ssrAB* expression. However, they were able to detect the positive regulatory effect of HilD on *ssrAB* under different conditions. They were also able to detect a direct interaction of both HilD and H-NS with *ssrAB*. Accordingly, they concluded that HilD activates SPI-2 gene expression via relieving the repressing effect of H-NS on *ssrAB*.
